# Annelid Comparative Genomics and the Evolution of Massive Lineage-Specific Genome Rearrangement in Bilaterians

**DOI:** 10.1093/molbev/msae172

**Published:** 2024-08-14

**Authors:** Thomas D Lewin, Isabel Jiah-Yih Liao, Yi-Jyun Luo

**Affiliations:** Biodiversity Research Center, Academia Sinica, Taipei, Taiwan; Biodiversity Research Center, Academia Sinica, Taipei, Taiwan; Biodiversity Research Center, Academia Sinica, Taipei, Taiwan

**Keywords:** Annelida, bilateria, synteny, genome organization, chromosome, rearrangement, lineage-specific evolution

## Abstract

The organization of genomes into chromosomes is critical for processes such as genetic recombination, environmental adaptation, and speciation. All animals with bilateral symmetry inherited a genome structure from their last common ancestor that has been highly conserved in some taxa but seemingly unconstrained in others. However, the evolutionary forces driving these differences and the processes by which they emerge have remained largely uncharacterized. Here, we analyze genome organization across the phylum Annelida using 23 chromosome-level annelid genomes. We find that while many annelid lineages have maintained the conserved bilaterian genome structure, the Clitellata, a group containing leeches and earthworms, possesses completely scrambled genomes. We develop a rearrangement index to quantify the extent of genome structure evolution and show that, compared to the last common ancestor of bilaterians, leeches and earthworms have among the most highly rearranged genomes of any currently sampled species. We further show that bilaterian genomes can be classified into two distinct categories—high and low rearrangement—largely influenced by the presence or absence, respectively, of chromosome fission events. Our findings demonstrate that animal genome structure can be highly variable within a phylum and reveal that genome rearrangement can occur both in a gradual, stepwise fashion, or rapid, all-encompassing changes over short evolutionary timescales.

## Introduction

The arrangement of genomic DNA into individual chromosomes creates a dynamic landscape subject to continual reshaping through processes such as fusion, fission, inversion, and translocation (Schubert 2007; Lysák and Schubert 2013; Simakov et al. 2022). These structural alterations play pivotal roles in fundamental biological phenomena, including recombination (Rieseberg 2001; Näsvall et al. 2023; Yoshida et al. 2023), adaptation (Dunham et al. 2002; Coyle and Kroll 2007; Lowry and Willis 2010; Jones et al. 2012; Wellenreuther and Bernatchez 2018), speciation (Noor et al. 2001; de Vos et al. 2020; Augustijnen et al. 2024), disease (Lupski and Stankiewicz 2005), and ultimately the emergence of novel phenotypic traits. While some lineages exhibit remarkable conservation of genome structure over extended evolutionary timescales, others display striking divergence, with chromosomes rearranged in unpredictable patterns (Wang et al. 2017a; Simakov et al. 2022; Ivankovic et al. 2023; Martín-Zamora et al. 2023; Lin et al. 2024). Unraveling the evolutionary forces driving such lineage-specific scrambling of gene sets can provide valuable insights into the process of adaptation and the evolution of animal diversity.

The sequencing of genomes to chromosome scale has facilitated the development of new methods for comparing genome structure. In many species, orthologous genes have remained clustered on the same chromosomes for over half a billion years since the ancestor of bilaterian animals (Simakov et al. 2013, 2022). This conserved gene linkage, or macrosynteny, can be used to track orthologous chromosomes across highly divergent species and is emerging as a powerful tool for studying genome rearrangements. This technique has thus far largely been applied to long-range comparisons across metazoans (Simakov et al. 2013, 2022; Schultz et al. 2023; Zimmermann et al. 2023) or to compare very closely related species, such as *Acropora* corals (Locatelli et al. 2023) or cryptic species of the tunicate *Oikopleura dioica* (Plessy et al. 2024). Such studies have been highly fruitful, elucidating the genome structure of the last common ancestor of bilaterians (Putnam et al. 2007, 2008; Simakov et al. 2022; Marlétaz et al. 2023) and developing algorithms for inferring rearrangement history (Ferretti et al. 1996; DasGupta et al. 1997; Mackintosh et al. 2023a, 2023b). Although several works have studied interchromosomal rearrangements across a phylum, including the chordates (Simakov et al. 2020; Huang et al. 2023) and hemichordates (Lin et al. 2024), most use few representative species and there is a lack of densely sampled phylum-wide studies. Key questions persist, including the degree of macrosynteny conservation within phyla, the frequency of large-scale reorganization events at lower taxonomic levels, the relative significance of fusion versus fission events, and the manner in which rearrangements unfold—whether gradually and stepwise or through sweeping changes over relatively short timescales.

The phylum Annelida represents a promising model to answer such questions. At the onset of this project, 24 chromosome-level annelid genomes were available from 15 families, including the basal owneniids, offering a data set with both depth and breadth of sampling. Annelids form a diverse group of segmented, vermiform (worm-like) spiralians that is split into two main clades, Errantia and Sedentaria, based on the dominant lifestyle of their members (i.e. errant or sedentary) (Bleidorn et al. 2015). Annelid species such as the bristle worm *Capitella teleta*, the ragworm *Platynereis dumerilii*, and the leech *Helobdella robusta* have emerged as key model systems, especially for questions surrounding the evolution of development (Weisblat and Kuo 2009; Kutschera and Weisblat 2015; Seaver 2016; Özpolat et al. 2021). While annelids are ancestrally ocean dwelling and the majority of annelid diversity remains marine, the subclass Clitellata, containing leeches and earthworms, made a highly successful foray into freshwater and terrestrial habitats (Bleidorn et al. 2015; Erséus et al. 2020). Notably, it has been reported from draft assemblies that conserved bilaterian chromosomes are present in *C. teleta* and the miniature annelid *Dimorphilus gyrociliatus* but not in the leech *H. robusta* or the earthworm *Eisenia andrei*, pointing toward the possibility of extensive chromosome rearrangements within this group (Simakov et al. 2013; Martín-Durán et al. 2021; Sun et al. 2021).

Here, we first produce gene annotations for 23 chromosome-level annelid genomes. Using these new gene models, we build an updated phylogeny of annelids and characterize annelid chromosome evolution. Our findings suggest that the last annelid common ancestor had a genome of 20 chromosomes, with four fusion events compared to the ancestor of bilaterians. This karyotype is deviated from only slightly within many annelid lineages, although fusion events are relatively frequent and have resulted in a chromosome number of <20 in all except one of the analyzed species. However, this conserved genome architecture has completely disintegrated within leeches and earthworms, and genes from conserved bilaterian linkage groups are shuffled across chromosomes. Using a newly defined rearrangement index, a metric aimed at quantifying the extent of chromosome rearrangement within genomes, we show that bilaterian genomes can be split into two categories, high or low rearrangement, and that the difference between them is largely driven by the incidence of chromosome fission events. Finally, we demonstrate that leeches and earthworms have among the highest levels of genome rearrangement of any bilaterian and suggest that this may have contributed to their derived morphology and adaptation to nonmarine environments.

## Results

### Phylogeny of Chromosome-Level Annelid Genomes

To study annelid genome evolution, we first assembled a data set of all public chromosome-level annelid genomes (supplementary table S1, Supplementary Material online). We performed gene prediction using RNA-sequencing (RNA-seq) data where available and protein data in its absence, producing a data set of 23 chromosome-level assemblies with highly complete gene models (supplementary fig. S1, Supplementary Material online). Genome size is highly variable among the sampled annelids, ranging from 149 Mb in the leech *Hirudinaria manillensis* to 1,861 Mb in the deep-sea hydrothermal vent scale worm *Branchipolynoe longqiensis* (mean assembly length 944 Mb) (supplementary fig. S2, Supplementary Material online). Across the sampled genomes, the mean GC content is 39.6% and the mean repeat content is 45.8%. The wide range of chromosome numbers, from 9 to 41 with a mean of 16, makes this data set particularly promising for studying interchromosomal rearrangements.

**Fig. 1.**
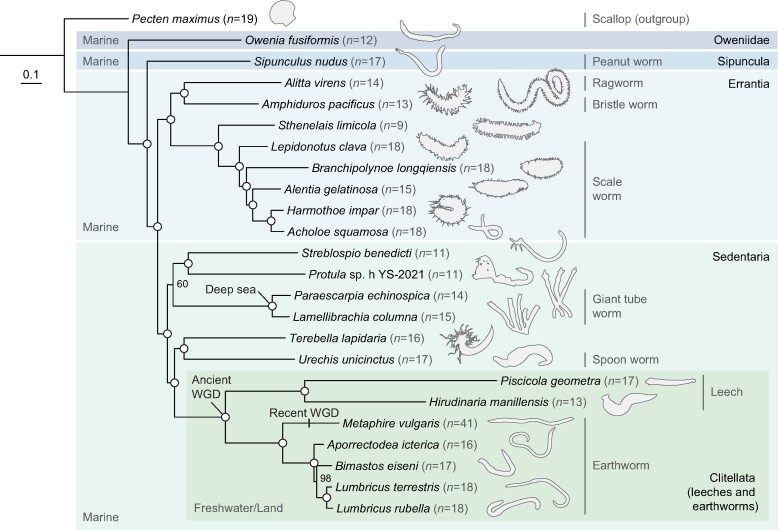
Phylogenomic analysis of annelids with chromosome-level genomes highlights lineage-specific evolution. The phylogenetic tree was constructed using the maximum likelihood method with the LG + F + R7 model based on a concatenated alignment of 537 single-copy orthologous protein sequences from 23 annelid genomes. The scallop *P. maximus* is used as an outgroup. Open circles represent a bootstrap score of 100% support. Numbers in parentheses show chromosome number.

Robust phylogenies form the foundations for understanding the direction of evolutionary change and are therefore a necessity for studying genome evolution. However, the current understanding of annelid phylogeny is largely based on transcriptomic data (Struck et al. 2011; Weigert et al. 2014; Andrade et al. 2015; Weigert and Bleidorn 2016) and subsequently retains a degree of uncertainty. We built a maximum likelihood phylogeny of annelids using the chromosome-level genomes and newly annotated gene models (Fig. 1; supplementary fig. S3, Supplementary Material online). The topology is largely consistent with transcriptome-based phylogenies and supports the widely accepted division of the bulk of annelid diversity into two monophyletic groups, Errantia and Sedentaria, with Oweniidae and Sipuncula as basal lineages. Within Sedentaria, Clitellata, including leeches and earthworms, forms a clade that is sister to a clade containing Echiuroidea (e.g. *Urechis unicinctus*) and Terebellida (e.g. *Terebella lapidaria*). This sister relationship of marine and freshwater clades highlights the lineage-specific evolution in habitat adaptation within the annelids.

### Bilaterian ALGs Are Often Fused But Rarely Split in Nonclitellate Annelids

**Fig. 2.**
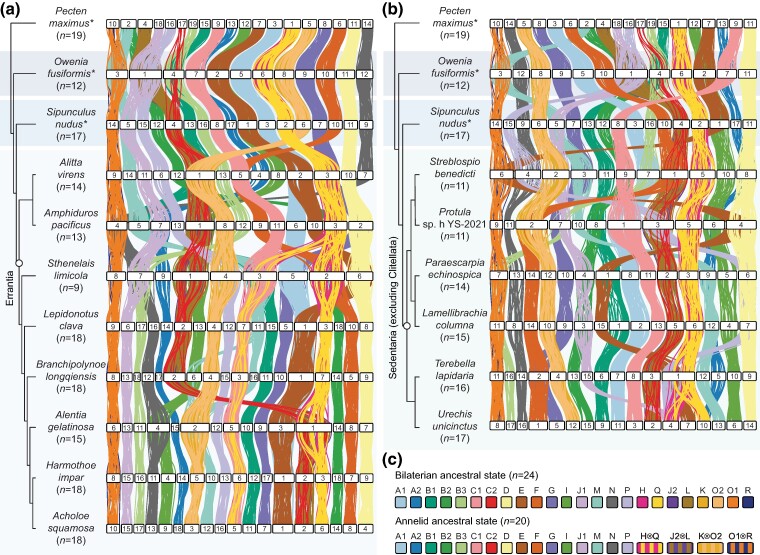
Bilaterian ALGs are often fused but rarely split in errantian and sedentarian annelids. Idiogram plots display the locations of shared single-copy orthologs on chromosomes in Errantia a) and Sedentaria b) annelids. Phylogenies show the relationships determined in Fig. 1, with the clades Errantia and Sedentaria highlighted with open circles. The scallop *P. maximus* and annelids *O. fusiformis* and *S. nudus* are used as outgroups and are labeled with asterisks. Horizontal white bars represent chromosomes. Vertical lines between species link orthologous genes; lines are colored by the bilaterian ALGs to which genes belong. c) The bilaterian ancestral state consisted of 24 linkage groups, while the annelid ancestral state had 20 linkage groups, with the following fusions compared to the bilaterian ancestral state: H⊗Q, J2⊗L, K⊗O2, and O1⊗R. The symbol ⊗ represents fusion-with-mixing events.

We next employed a macrosynteny approach to study interchromosomal rearrangements in annelids. To achieve this, we identified single-copy orthologs, assigned them to bilaterian ancestral linkage groups (ALGs) based on protein sequence homology, mapped their genomic locations, and used idiogram plots (Fig. 2) and Oxford dot plots (supplementary figs. S4 and S5, Supplementary Material online) to track chromosome relationships between species (supplementary table S2, Supplementary Material online). The last bilaterian common ancestor had 24 ALGs, sets of genes that were subsequently inherited by all bilaterian phyla (Simakov et al. 2022), and we first questioned whether these are conserved in annelids. We found that all 24 ALGs were present in annelids and identified four rearrangements shared by all annelids: H⊗Q, J2⊗L, K⊗O2, and O1⊗R, where the symbol ⊗ represents ALG fusion with mixing, indicating that genes from the fused chromosomes are mixed by intrachromosomal rearrangements (Simakov et al. 2022). From this, it can be inferred that the annelid ancestral state was 20 ALGs and, therefore, likely 20 chromosomes. Indeed, these four rearrangements are also shared by other lophotrochozoans, including molluscs, nemerteans, bryozoans, and brachiopods (Simakov et al. 2022; Lewin et al. 2024a), suggesting they are common to most or all lophotrochozoans. Therefore, there are no unique interchromosomal rearrangements shared by all annelids, and the last annelid common ancestor retained the ancestral lophotrochozoan karyotype with 20 chromosomes. There is a C2⊗(J2⊗L) fusion (where parentheses indicate ALGs already fused in the ancestor of annelids) in all sampled annelids except *Owenia fusiformis*, but there are no chromosome rearrangements that act as synapomorphies for either of the large annelid clades, Errantia or Sedentaria (Fig. 2; supplementary fig. S6, Supplementary Material online).

In addition to the four chromosome fusion events present in all annelids, there are many further chromosome rearrangements restricted to specific lineages. The genomes of all sampled species have at least three ALG fusions compared to the ancestral annelid genome. There is only one case of two species sharing an identical genome structure in this data set, *Harmothoe impar* and *Acholoe squamosa* (*n* = 18), which are both within the same family (Polynoidae). *Sthenelais limicola* (*n* = 9) has the highest number of fusions (12) while *Sipunculus nudus* (*n* = 17), *Lepidonotus clava* (*n* = 18), *H. impar* (*n* = 18), and *A. squamosa* (*n* = 18) have the fewest (3) (supplementary fig. S7, Supplementary Material online). We find that almost all the chromosome fusion events in annelids can be categorized as fusion with mixing, where ALG fusion is followed by shuffling and interspersal of genes from the fused ALGs. There are only three putative fusion events without mixing, where genes from the fused ALGs remain separate on the fused chromosome (notation ●): B1●E on *Paraescarpia echinospica* chromosome 1; (H⊗Q)●(E⊗P) on *T. lapidaria* chromosome 1; and G●(B3⊗J1) on *Protula* sp. h YS-2021 chromosome 7. All other events can be characterized as fusion with mixing.

A recent study in Lepidoptera (butterflies and moths) found that ALGs' propensity for fusion was inversely correlated with the length of the chromosomes on which they reside (Wright et al. 2024). In annelids, we found no correlation between the ALG fusion rate and the length of chromosomes (Spearman's rank correlation coefficient = 0.125, *P* = 0.607) (supplementary fig. S8 and tables S3 and S4, Supplementary Material online). Indeed, there was no significant difference in the rates at which ALGs fused (*χ*^2^ test, *P* = 0.736), suggesting that in nonclitellate annelids, certain ALGs are not more prone to fusing than others.

In contrast to fusions, the splitting of ALGs is relatively rare, with only three cases in the 16 nonclitellate species. The ALG H⊗Q is split independently in the suborder Aphroditiformia and in *U. unicinctus*, and the ALG M is split in *B. longqiensis*. We note that ALG H⊗Q is housed on the second-longest chromosomes in the data set, but more data are needed to determine whether ALG splitting is associated with chromosome length. In all cases, ALG splitting coincides with the fusion of part of an ALG to another chromosome rather than simple chromosome fission. Overall, ALG fusion is very common but ALG fission is rare in nonclitellate annelids.

### Total Loss of Bilaterian Genome Structure in Clitellates, the Group Containing Leeches and Earthworms

**Fig. 3.**
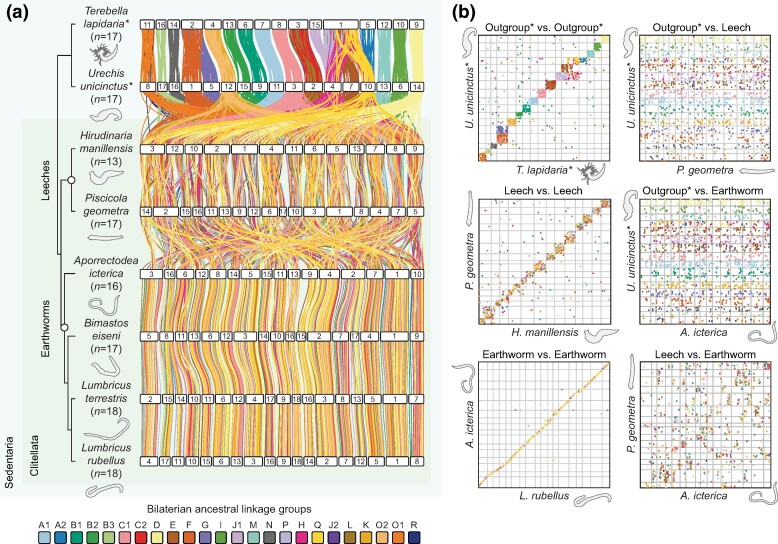
Bilaterian ALGs are completely rearranged in leech and earthworm genomes. a) Synteny analysis of clitellate annelid genomes using idiograms. Horizontal white bars represent chromosomes. Vertical lines between species connect orthologous genes and are colored according to the bilaterian ALGs to which the genes belong. Phylogeny reflects the topology determined in Fig. 1. The nonclitellate annelids *T. lapidaria* and *U. unicinctus* are utilized as outgroups and are marked by asterisks. b) Oxford dot plots showing the position of orthologous genes in pairs of annelid genomes. Dots organized into quadrangles indicate the conservation of macrosynteny (genes on the same chromosome) but not microsynteny (gene order on the chromosome), as seen in comparisons within the outgroup. In contrast, dots aligned in a straight line represent the conservation of both macrosynteny and microsynteny, such as in earthworm versus earthworm comparisons. Dots scattered randomly across the plot without any clear organization suggest no conservation of macrosynteny or microsynteny.

Chromosome evolution in the species considered above is characterized by the broad maintenance of bilaterian ALGs with relatively frequent lineage-specific fusion events. Clitellata, including leeches and earthworms, is a morphologically divergent monophyletic group of annelids nested within the Sedentaria. Performing macrosynteny analysis on the genomes of six clitellates, we found that bilaterian ALGs have been completely lost in this clade (Fig. 3a). Remarkably, further synteny analysis using dot plots reveals that in both leeches and earthworms, there is complete shuffling of the ancient bilaterian genome, with each ALG spread across all chromosomes (Fig. 3b). This stands in clear contrast to nonclitellate annelids, where ancestral bilaterian chromosomes have been retained with high fidelity. Interestingly, while genome structure is largely conserved within the leeches and earthworms, there has also been massive genome shuffling between these two groups. Their genomes cannot be easily mapped to each other and have highly divergent organizational structures (Fig. 3b). Overall, the ancient bilaterian genome architecture has been completely lost within the clitellates.

### Lineage-Specific WGD in Earthworms

**Fig. 4.**
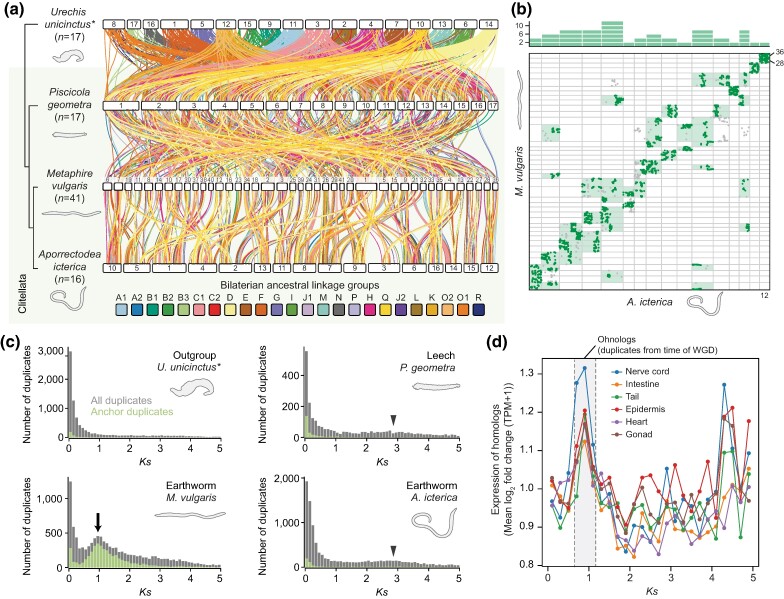
Organization of bilaterian ALGs and synonymous substitution rate support lineage-specific WGDs in clitellates. a) Idiogram plot of *M. vulgaris* (earthworm) genome with those of *A. icterica* (earthworm), *P. geometra* (leech), and *U. unicinctus* (outgroup). Horizontal bars represent chromosomes. Vertical lines between species link orthologous genes; lines are colored by the bilaterian ALG to which genes belong. Phylogeny reflects the topology determined in Fig. 1. The *M. vulgaris* genome is completely rearranged compared to Sedentaria species and leeches but shows some conserved macrosynteny with other earthworms. Many areas of the earthworm *A. icterica* genome correspond to two areas of the *M. vulgaris* genome. Links between chromosomes with fewer than 20 orthologous genes are trimmed from the plot for clarity. b) Oxford dot plot of *M. vulgaris* and *A. icterica* genomes, where each point represents an orthologous gene's position in both genomes. Plot shows single-copy orthologs and one-to-many orthologs with up to five copies in one species. Sets of orthologues with a 1:2 ratio in *A. icterica* versus *M. vulgaris* are shown in dark green. For instance, *A. icterica* chromosome 12 corresponds to *M. vulgaris* chromosomes 28 and 36, with the chromosomes arranged in the same order as in a). The bar chart at the top shows the number of cases of a clearly identifiable 1:2 ratio with *M. vulgaris* on each *A. icterica* chromosome. Boxes representing the chromosomes on which they appear are highlighted in light green. The plot shows that many chromosome sections in *A. icterica* correspond to two chromosome sections in *M. vulgaris*, suggestive of recent WGD. A larger version of this figure with chromosomes labeled is presented as supplementary fig. S10, Supplementary Material online. c) *K_S_* plots for duplicated gene families. Gray bars show histograms for all duplicated genes, while green bars show only anchor duplicates: these are duplicated genes found in duplicated collinear blocks of genes in the genome. Genomes with no WGD are expected to show exponential decay of duplicate numbers as *K_S_* increases, as seen in the *U. unicinctus* (outgroup) plot. The *M. vulgaris* plot is interrupted by a large normally distributed peak at *K_S_* = 1, indicating recent WGD (arrow). The plots of the leech *P. geometra* and the earthworm *A. icterica* display a broad, shallow peak between *K_S_* = 2 and *K_S_* = 4, suggestive of ancient WGD(s) (arrowheads). d) Difference of expression of *M. vulgaris* gene duplicate pairs (measured in log_2_ fold change of transcripts per million + 1) in six tissues plotted against *K_S_*, a proxy for time since duplication. Note that there is a peak of expression difference in gene duplicate pairs that emerged at the time of the recent WGD in all six tissues.

Given the massive genome shuffling present in all clitellates, we questioned whether a whole-genome duplication (WGD) reported in *Metaphire vulgaris* (Jin et al. 2020) is present across other leeches and earthworms. Our bilaterian ALG-based macrosynteny approach suggests that the recent duplication is unique to *M. vulgaris*: we found that macrosynteny is partially conserved with other earthworms like *Aporrectodea icterica* but there is a 1:2 correspondence between many sections of the *A. icterica* genome with that of *M. vulgaris*. This 1:2 ratio is visible both in an idiogram plot (Fig. 4a) and Oxford dot plot (Fig. 4b; supplementary figs. S9 and S10, Supplementary Material online) and is highly suggestive of a recent WGD in *M. vulgaris* but not *A. icterica*.

To examine the presence of WGDs in clitellates more closely, we produced synonymous substitution rate (*K_S_*) plots for gene duplicates within each genome. *K_S_* measures the number of synonymous substitutions per site and, assuming that such changes are neutral and occur at a constant rate, estimates the evolutionary time since a gene duplication (Maere et al. 2005). In typical genomes with no WGD, the largest number of retained duplicates is evolutionarily young with low *K_S_*, and the number of retained duplicates decreases exponentially as *K_S_* increases. However, a WGD produces many duplicates at the same time, resulting in an excess of gene families of the same age; this manifests as a normally distributed peak at an intermediate *K_S_* value (Blanc and Wolfe 2004; Schlueter et al. 2004; Tiley et al. 2018).

We found a large peak at *K_S_* = 1 in the plot for *M. vulgaris* but not for leeches, other earthworms, or more distantly related annelids (Fig. 4c; supplementary fig. S11, Supplementary Material online). This strongly supports the above conclusion of a recent WGD in *M. vulgaris*. Furthermore, the pattern is also repeated when only anchor gene duplicates are considered. Anchors are genes that are present in duplicated collinear blocks in the genome (i.e. conserved microsyntenic blocks), suggesting that they are not tandem duplicates and more likely arose from WGD events (Tang et al. 2008; Myburg et al. 2014). This makes them more reliable for constructing *K_S_* plots; the presence of an anchor gene *K_S_* peak in *M. vulgaris* but not other species therefore supports a recent WGD in this lineage (Fig. 4c; supplementary fig. S11, Supplementary Material online). We further confirmed the recent WGD by analyzing the locations of these anchor duplicates within the assembled scaffolds, which revealed large collinear strings of duplicated genes in *M. vulgaris* that are absent in other annelids (supplementary figs. S12 and S13, Supplementary Material online). In addition to the recent WGD in *M. vulgaris*, there is evidence of an ancient WGD in the evolutionary history of clitellates. In all sampled leeches and earthworms, a broad, shallow peak between *K_S_* values of 2 and 4 suggests the possibility of a relatively ancient WGD (Fig. 4c; supplementary fig. S11, Supplementary Material online). In contrast, there is no evidence of WGD in nonclitellate annelids. Overall, these findings suggest the presence of an ancient WGD at the base of the clitellates.

A key question emerging from these results is what the biological effects of these genomic changes might be. We questioned whether gene duplicates formed by the WGD evolve differently to those formed by non-WGD duplication events. To answer this, we plotted duplicate pairs' *K_S_* values against the log_2_ fold difference in their expression level in RNA-seq data sets from six *M. vulgaris* tissues (supplementary tables S5 and S6, Supplementary Material online). We found a peak in the difference of expression in duplicate pairs that emerged at the time of the recent WGD (Fig. 4d). This result suggests that duplicate genes derived from the recent WGD diverged in expression more quickly than those formed by other processes, such as tandem duplication, underlining the potential importance of WGD events to evolvability and adaptation.

### Exceptional Levels of Genome Reorganization in Clitellates

Next, we investigated whether the level of genome rearrangement observed in clitellates is unusual within the context of other bilaterians. To test this, we developed a macrosynteny rearrangement index, *R_i_* (see Materials and Methods; and see supplementary fig. S14, Supplementary Material online for a thorough explanation), a metric to quantify both ALG fusion and fission into a single value between 0 (no rearrangement) and 1 (maximum rearrangement). The index considers macrosynteny (chromosomal colocalization) alone and does not consider microsynteny (conserved gene order). Unlike a previous conservation index (Simakov et al. 2013), it does not require pairwise comparisons and can therefore be computed for a single genome and does not rest on potentially unreliable homology of chromosomes or scaffolds. The rearrangement index comprises an ALG splitting parameter (*S*_CHR_) and an ALG combining parameter (*C*_CHR_). Although we expect the combining parameter to largely reflect ALG fusion and the splitting parameter to largely reflect ALG fission, the index cannot distinguish fusion and fission from other mechanisms, such as reciprocal translocation, so in the below discussion, we avoid the terms fusion and fission and instead use splitting (genes from one ALG being separated on to different chromosomes) and combining (genes from different ALGs coming together on the same chromosome).

**Fig. 5.**
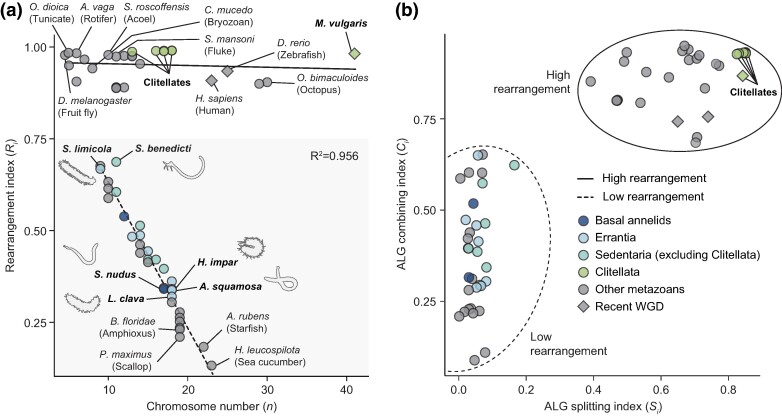
Clitellate annelids have exceptional levels of interchromosomal rearrangements among bilaterians. a) Rearrangement index versus chromosome number in bilaterian genomes. Rearrangement index measures bilaterian ALG splitting and combining: higher indices reflect more rearrangement, and lower indices show less rearranged genomes. Lineages with recent WGDs are marked with a diamond. The relationships between rearrangement index and chromosome number were examined using linear models for high (solid line) and low (dashed line) rearrangement groups. The estimated model for low rearrangement species *y* = −0.040*x* + 1.032 has a statistically significant slope (standard error = 0.002, *P* = 7.378 × 10^−23^), suggesting that the rearrangement index increases linearly as the chromosome number decreases. The *R*^2^ value of 0.956 suggests that ∼96% of the variation in rearrangement index in these species is explained by chromosome number. The estimated model for high rearrangement species *y* = −0.0004*x* + 0.958 has a nonsignificant slope (standard error = 0.001, *P* = 0.659), suggesting that chromosome number does not predict rearrangement index in these species. The *R*^2^ value of −0.029 suggests that no variation in the rearrangement index is predicted by chromosome number. b) ALG combining index versus ALG splitting index in bilaterian genomes. See Materials and Methods for full description of the calculation of these parameters. Higher index values indicate increased levels of rearrangement.

We used the rearrangement index to compare rearrangement levels in our annelid data set with 39 additional species from 12 other bilaterian phyla for which chromosome-level assemblies were available (Fig. 5a; supplementary fig. S15 and table S7, Supplementary Material online). We first validated our approach by studying species previously reported to have highly rearranged genomes. The high rearrangement indices of the tunicate *O. dioica* (*n* = 5) (Denoeud et al. 2010; Plessy et al. 2024), the octopus *Octopus bimaculoides* (*n* = 30) (Albertin et al. 2022), the fruit fly *Drosophila melanogaster* (*n* = 4) (Wang et al. 2017a), the freshwater bryozoan *Cristatella mucedo* (*n* = 8) (Lewin et al. 2024a), and the blood fluke *Schistosoma mansoni* (*n* = 11) (Wang et al. 2017a; Ivankovic et al. 2023) demonstrate that the index correctly identifies these species as highly rearranged. Conversely, deuterostomes such as the starfish *Asterias rubens* (*n* = 22) and the sea cucumber *Holothuria leucospilota* (*n* = 23) have the lowest levels of rearrangement, maintaining genome structures most similar to that of the bilaterian ancestor. The scallop *Pecten maximus* (*n* = 19) and the amphioxus *Branchiostoma floridae* (*n* = 19) also have highly conserved genomes with minimal rearrangement. Other species that we identified with highly rearranged genomes include fast-evolving lineages with few chromosomes like the rotifer *Adineta vaga* (*n* = 6) and parasites like the nematomorph *Gordionus* sp. RMFG-2023 (*n* = 5) and the symbiotic acoel *Symsagittifera roscoffensis* (*n* = 10). Strikingly, clitellate annelids have among the highest rearrangement indices of all sampled bilaterians.

Interestingly, we found that bilaterian genomes fell into one of two groups, a high rearrangement group and a low rearrangement group, which are separated on the plot (Fig. 5a; supplementary fig. S15, Supplementary Material online). We used linear models to examine the relationship between rearrangement index and chromosome number for each of the two groups. This showed that, in the low rearrangement group, over 95% of the variation of rearrangement index was explained by chromosome number (*P* = 7.378 × 10^−23^, *R*^2^ = 0.956). Indeed, rearrangement index increases linearly as chromosome number decreases from the bilaterian ancestral state of 24 gene linkage groups. This suggests that the index largely reflects chromosome fusion. Nonclitellate annelids conform to this pattern: for instance, *L. clava* (*n* = 18) has the lowest rearrangement index and *Streblospio benedicti* (*n* = 11) the highest. In contrast, the chromosome number is not predictive of rearrangement index in the high rearrangement group (*P* = 0.659, *R*^2^ = −0.029). This result suggests that there are two separate groups of bilaterians in which different patterns of interchromosomal rearrangements are observed. It is remarkable that there has been a distinct shift in the mode of chromosome rearrangement within annelids: clitellates are part of the high rearrangement group, while nonclitellates fall into the low rearrangement group.

To better understand the factors distinguishing these two groups, we separated out the bilaterian ALG combining and ALG splitting parameters (Fig. 5b; supplementary fig. S16, Supplementary Material online). Our analysis reveals that both ALG combining and ALG splitting are higher in the high rearrangement group but that there is a large gap in the ALG splitting index specifically. Low rearrangement species have minimal ALG splitting (splitting index < 0.17), while members of the high rearrangement group all have a splitting index in excess of 0.39. Based on the annelid genomes, we can infer that the difference between these two groups is the absence of chromosome fission in the low rearrangement species. To further understand the underlying causes of this distinction, rather than averaging the index across all ALGs, we performed a principal component analysis (PCA) using the individual index scores for each ALG in each species (supplementary fig. S17 and table S8, Supplementary Material online). The high rearrangement and low rearrangement groups were well separated in the PCA. The dimension distinguishing the two groups (PC1) accounted for over 75% of the variance in the data set, and the top 20 variables contributing to this dimension were all ALG splitting indices (supplementary fig. S17, Supplementary Material online). This confirms that the major factor separating the high and low rearrangement groups is the extent of ALG splitting. In turn, this suggests that there are two groups of bilaterian genomes, those in which ALG fission is evolutionarily permitted and frequent and those in which it is highly restricted.

Intriguingly, we observed that each of the 11 smallest genomes falls into the high rearrangement group, pointing to an association between genome size and the extent of interchromosomal rearrangement. These 11 assemblies are drawn from seven phyla, suggesting that this is not simply the product of a phylogenetic bias caused by one clade with small, rearranged genomes. Across the whole data set, we detected no significant difference between the genome size of high versus low rearrangement species (two-tailed unpaired *t*-test; *P* = 0.105, standard error of difference = 149 Mb) (supplementary fig. S18, Supplementary Material online). However, this may be a result of biases in the sampling of genomes. Consistent with this, we found that clitellate genomes are significantly smaller than those of nonclitellate annelids (two-tailed unpaired *t*-test; *P* = 0.030, standard error of difference = 170 Mb). We also noted that clitellates have a higher rate of protein sequence evolution than nonclitellate annelids (supplementary fig. S19, Supplementary Material online). This, combined with the observation that other high rearrangement lineages such as *O. dioica* are known to be rapidly evolving (Berná et al. 2012), suggests that the rates of sequence evolution and interchromosomal rearrangement may be correlated. Overall, these results hint at an association of genome size and the rate of evolutionary sequence change with the propensity for interchromosomal rearrangement, but further data are necessary to test this hypothesis.

### Macrosynteny as a Tool for Taxonomy within a Phylum

**Fig. 6.**
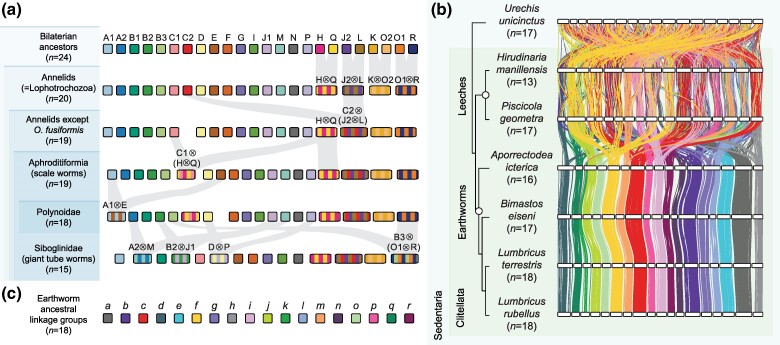
Conservation of interchromosomal rearrangements defines distinct taxonomic groups. a) Stepwise evolution of genome reorganization events in Annelida. Boxes represent linkage groups or chromosomes colored by their bilaterian ancestral state. Fusion-with-mixing events are represented by striped chromosomes. Interchromosomal rearrangements that are diagnostic for specific lineages are highlighted with gray ribbons. Shaded areas on the left surrounding taxon names represent progressively smaller subsets of species. For instance, Polynoidae is a subset of Aphroditiformia and Aphroditiformia is a subset of “Annelids except *O. fusiformis*.” b) Idiogram plots of clitellate genomes colored by earthworm ALGs. Earthworm ALGs are defined by genes' position in *L. rubellus.* c) Color code for earthworm ALGs. These are highly conserved across the four earthworm species in this data set.

The past year has seen macrosynteny emerge as a novel tool for delineating phylogenetic relationships (Parey et al. 2023; Schultz et al. 2023; Lewin et al. 2024a; Steenwyk and King 2024). In particular, chromosome fusion-with-mixing events have significant potential as phylogenetically informative rare genomic changes (i.e. molecular synapomorphies) because they are irreversible (Rokas and Holland 2000; Schultz et al. 2023; Steenwyk and King 2024). We used our data set to test the power of ALG-based macrosynteny as a taxonomic tool by asking whether it can be used to reliably identify characteristics for defining monophyletic groups of annelids. Within the data set of 16 nonclitellate species, we found four clades with lineage-defining interchromosomal rearrangements. First, all annelids except the basal oweniid *O. fusiformis* have a C2⊗(J2⊗L) fusion; second, within Errantia, the suborder Aphroditiformia (scale worms) is defined by a C1⊗partial_(H⊗Q) fusion; third, members of the family Polynoidae share a further A1⊗E fusion; and fourth, the Sedentaria family Siboglinidae (giant tube worms) shares four fusions (A2⊗M, B2⊗J1, B3⊗(O1⊗R), and D⊗P) (Fig. 6a). We noted that several of these changes occur in a stepwise fashion, leading to progressively more derived genomes. For instance, all sampled annelids except *O. fusiformis* have C2⊗(J2⊗L); within this group, Aphroditiformia annelids have C1⊗partial_(H⊗Q); then, within Aphroditiformia, the Polynoidae has A1⊗E; and within Polynoidae, there are species-specific changes (e.g. I⊗N, M⊗(K⊗O2), and partial_(H⊗Q)⊗(C2⊗(J2⊗L) in *Alentia gelatinosa*).

These clade-defining rearrangements reveal the potential of ALG-based macrosynteny for within-phylum systematics. For instance, the absence of the C2⊗(J2⊗L) fusion in *O. fusiformis* and its presence in *S. nudus* unequivocally confirms the Oweniidae as a basal annelid lineage and Sipuncula as an annelid despite its lack of segmentation and appendages, more closely related to Errantia and Sedentaria than to *O. fusiformis*. This result demonstrates the ability of interchromosomal rearrangements to be used as taxonomically informative characters.

To facilitate macrosynteny comparisons in the completely rearranged clitellate genomes, we assigned newly formed but subsequently conserved gene groups to ALGs. Determination of earthworm (Fig. 6b and c; supplementary fig. S20, Supplementary Material online) and leech (supplementary fig. S21, Supplementary Material online) ALGs reveals strong conservation of macrosynteny within each group but also partial conservation of ALGs shared across both taxa. Therefore, leeches and earthworms can be defined by their own distinct ALGs, and the two groups share a recent common ancestor in which significant genome rearrangement had already occurred. Overall, in this data set alone, at least seven monophyletic groups (nonbasal annelids, Aphroditiformia, Polynoidae, Siboglinidae, Clitellata, leeches, and earthworms) can be defined by specific interchromosomal rearrangements that may be used for taxonomic classification (supplementary table S9, Supplementary Material online).

## Discussion

Our study reveals two distinct groups of annelids: nonclitellates, in which genome organization is characterized by broad conservation of the ancestral bilaterian genome architecture followed by limited chromosome fusions; and clitellates, in which the genome has been shuffled to such an extent that bilaterian ALGs have been completely lost. Indeed, this is a microcosm of the situation within bilaterians as a whole. By quantifying the extent to which genomes are rearranged, we found that bilaterians, like annelids, fall into one of two categories: low rearrangement, typically with some ALG fusion but low levels of fission, and high rearrangement, in which both fusion and fission are common. This supports the hypothesis that there is an evolutionary constraint on genome structure (present in nonclitellates), which can be flicked off like a switch (e.g. clitellates), causing rapid, extensive genome rearrangement and the complete disintegration of previously conserved ALGs.

Our findings imply that switches from conservative evolution of genome structure to rapid rearrangement, including ALG fission, have occurred independently on many occasions throughout bilaterian evolution. In addition to annelids, there are molluscs and chordates in both the high and low rearrangement categories, demonstrating convergent transitions to a state in which large genome rearrangements are evolutionarily favored or tolerated. Indeed, even with the current limited availability of chromosome-level assemblies, species from ten phyla are present in the high rearrangement group (i.e. Annelida, Arthropoda, Bryozoa, Chordata, Mollusca, Nematoda, Nematomorpha, Platyhelminthes, Rotifera, and Xenacoelomorpha), suggesting that massive genome scrambling is a widespread phenomenon across bilaterians.

The key question is what selective pressures control the switch from the conservative evolution of ALGs to their complete atomization in specific clades. Given that clitellates possess some of the highest rearrangement indices of all species in our data set, they may be an optimal group to investigate this question. We consider three potential triggers that warrant further consideration: WGD, incapacitation of DNA repair pathways, and transposable element invasion. First, we identified a putative ancient WGD shared by all clitellates and a lineage-specific recent WGD in *M. vulgaris*. This initial WGD coincides with the clitellate-specific atomization of genome structure and may have contributed to their genome instability. Indeed, studies from teleosts identified brief periods of genome rearrangement immediately following WGD (Kasahara et al. 2007; Sémon and Wolfe 2007a; Sémon and Wolfe 2007b; Kuraku et al. 2024), suggesting that this phenomenon may have also occurred in clitellates. However, many highly rearranged species, such as octopuses (Albertin et al. 2015), blood flukes (Wang et al. 2017b), and lepidopterans (Nakatani and McLysaght 2019), show no signs of WGD. Therefore, as WGD is clearly not a universal driver of the transition from low to high levels of genome rearrangement, it may not have been involved in clitellates. Second, loss of DNA repair machinery is known to increase rates of interchromosomal rearrangement (Bohlander and Kakadia 2015). The high rearrangement genomes of *O. dioica* (Deng et al. 2018), *D. melanogaster* (Sekelsky 2017), and clitellates (Vargas-Chávez et al. 2024) do exhibit such losses. However, like WGD, this is not common to all transitions to a high rearrangement state (Jackson and Bartek 2009) and is therefore also unlikely to be a widely applicable explanation.

Another possible contributor is the action of transposable elements. Transposons have long been known as powerful drivers of genome instability and chromosomal rearrangements, for instance, by facilitating ectopic recombination through the dispersal of homologous sites around the genome (Evgen’ev et al. 2000; Hill et al. 2000; Oliver and Greene 2009). Indeed, a recent study utilizing a macrosynteny approach found that higher transposon density is associated with increased rates of chromosome fusion in Lepidoptera (butterflies and moths) (Wright et al. 2024). The ability of transposons for rapid invasion and expansion (Kofler et al. 2018) is an attractive explanation for the sudden switches from conservative genome evolution to massive chromosomal instability. As an exploratory analysis, we annotated transposable elements in the annelid assemblies and compared the overall repeat content in clitellate and nonclitellate assemblies and found no significant differences (supplementary fig. S22, Supplementary Material online). However, future studies need to consider specific repeat families, aim to test more generally whether the evolutionary timing of genome rearrangement correlates with repeat expansion, and, importantly, ask whether there are consistent differences between high and low rearrangement groups.

How do chromosome rearrangements spread and become fixed in a lineage? They may be adaptive and favored by positive selection or neutral/deleterious and spread by genetic drift (Wright 1941; Bush et al. 1977; Lande 1979; Mackintosh et al. 2023a, 2023b). Drastic rearrangements like those observed in clitellates are likely to be detrimental to fitness as heterozygotes (known as underdominance), as chromosome pairing at meiosis will be compromised (White 1973; Faria and Navarro 2010). Additionally, long-range interactions between distant chromosome regions are crucial for gene regulation (Tolhuis et al. 2002; Miele and Dekker 2008; Dean 2011). Chromosome-scale rearrangements that disrupt these interactions can interfere with the function of regulatory elements and consequently affect gene expression (Harewood and Fraser 2014; Spielmann et al. 2018). Moreover, due to the importance of chromosome 3D spatial positioning within the nucleus, rearrangements that cause physical repositioning of chromosomes can have a knock-on effect, modifying gene expression not only on the chromosomes involved but on many other chromosomes throughout the genome (Harewood et al. 2010; Di Stefano et al. 2020). Widespread disruption to gene expression appears unlikely to have a positive effect on fitness in most scenarios, and accordingly, a recent study in *Brenthis* butterflies found that most rearrangements are fixed by drift, indicating that they are neutral or weakly deleterious (Mackintosh et al. 2023a, 2023b). If this holds true, periods of small effective population size, population bottlenecks, inbreeding, or population structure, which increase genetic drift and reduce the impact of underdominance (Lande 1979; Walsh 1982), may have contributed to the fixation of massive rearrangements in the clitellate lineage. However, evidence for a selective sweep at the site of one chromosome fusion event in butterflies (Mackintosh et al. 2023a, 2023b) and multiple fusion sites in copepods (Du et al. 2024) suggests that positive selection could also contribute.

Previous studies have noted that the loss of bilaterian genome structure in clitellates coincides with the transition from marine to freshwater and terrestrial environments (Rousset et al. 2008; Erséus et al. 2020; Sun et al. 2021). Indeed, this process was associated with a high degree of morphological and life-history evolution in clitellates, including the presence of cocoon-producing clitellum, the evolution of direct development, and an increase in the frequency of parthenogenesis (Kuo 2017; Pandian 2019). Additionally, while most nonclitellate annelids are gonochores with distinct male and female sexes, the majority of clitellates are hermaphrodites (Pandian 2019). Genome rearrangement may be selectively favored during adaptation to a radically different environment because it facilitates changes to regulatory landscapes and therefore novel gene expression patterns. Consistent with this, Hox genes, the expression of which is critical for early development and highly dependent on genome organization (Duboule 2007; Rekaik and Duboule 2024), are extensively rearranged in the genomes of the earthworms *Eisenia fetida* and *Perionyx excavatus* and the leech *H. robusta* (Cho et al. 2012; Simakov et al. 2013; Zwarycz et al. 2015; Barucca et al. 2016). Importantly, there are data suggesting that Hox expression is divergent in leeches compared to other annelids (Kourakis and Martindale 2001; Gąsiorowski et al. 2023), but a more comprehensive analysis is needed to confirm this hypothesis. Overall, dramatic genome rearrangement in clitellates correlates with the evolution of a new ecological niche, alongside divergent genomic location and altered expression of key developmental genes.

Our phylum-level data set is of sufficient depth to start to identify trends in ALG evolution. First, it reveals that species within a phylum can have completely divergent genome structures, with bilaterian ALGs preserved with high fidelity in some and completely lost in others. Second, it shows that interchromosomal rearrangements can occur both in a gradual stepwise fashion (nonclitellates) and as rapid, sweeping changes (clitellates). Third, ALG fusion is almost always followed by ALG mixing within the chromosome, and fusion without mixing is very rare. Fourth, fusion of ALGs is much more common than fission, suggesting strong selective pressures to maintain genes together on the same chromosome. This is supported by data from Lepidoptera, which also revealed fission to be much less common than fusion (Wright et al. 2024).

While methods for phylogeny reconstruction using microsynteny (small-scale conservation of gene order) are becoming increasingly sophisticated (Drillon et al. 2020; Zhao et al. 2021), the use of ALG-based macrosynteny for phylogenetic inferences is in its infancy. In general, although not without exception (Li et al. 2022), macrosynteny appears to decay slower than microsynteny (Simakov et al. 2022), meaning that it may have a unique utility for delineating relationships between distantly related groups. Recent works placing ctenophores as the basal metazoan lineage (Schultz et al. 2023), suggesting that bryozoans are closely related to brachiopods (Lewin et al. 2024a) and resolving branching order in teleost fishes (Parey et al. 2023), highlight its significant potential. Within this data set of 23 annelids, we describe unique chromosome rearrangements that can be used as rare genomic changes to define seven different taxonomic groups at levels varying from class to family. For example, genome structure definitively supports Sipuncula (in the past considered a separate phylum) as an annelid and Oweniidae as a basal annelid lineage due to the presence of the C2⊗(J2⊗L) fusion in the former and absence in the latter, confirming data from sequence-based phylogenetics (Weigert et al. 2014; Struck et al. 2015; Zhong et al. 2022; Zheng et al. 2023). Importantly, the observed stepwise manner of ALG rearrangements suggests that changes to genome structure as clade-defining characters need not be restricted to a specific taxonomic level but can be applied at any level from metazoan wide to genus and species. At present, the sampling depth is likely to limit the utility of this to a few, specific cases, but the accelerating accumulation of chromosome-level assemblies makes it inevitable that, in the coming years, many groups will have sufficiently dense sampling for robust genome structure-based taxonomic definitions.

One strength of this framework is its potential for disentangling the evolutionary relationships between fast-evolving lineages. We propose that rapidly evolving genomes like those of clitellates, while troublesome for sequence-based phylogenetics due to artifacts like long-branch attraction (Felsenstein 1978; Bergsten 2005), may be ideal for genome structure-based taxonomy due to the rapid accumulation of genome rearrangements and the improbability that highly complex rearrangements could be convergently evolved. Therefore, genome structure-based taxonomy may be particularly helpful for elucidating the positions of traditionally problematic lineages.

## Materials and Methods

### Assembly Acquisition and Gene Prediction

This study aimed to characterize interchromosomal rearrangements within the phylum Annelida. All available chromosome-level assemblies of annelids, representing 24 species, were obtained from the National Center for Biotechnology Information (NCBI) using NCBI Datasets on February 1, 2024. Of the 24 genomes, 16 were produced by the Darwin Tree of Life (DToL) sequencing project (The Darwin Tree of Life Project Consortium et al. 2022). The genome assemblies from the DToL project are made publicly available to the community. Those with an accompanying publication are *A. squamosa* (Adkins, Brennan, et al. 2023), *A. gelatinosa* (Adkins, Mrowicki, et al. 2023), *Alitta virens* (Fletcher et al. 2023), *Bimastos eiseni* (Brown et al. 2024), *H. impar* (Adkins, Mrowicki, Harley, et al. 2023), *L. clava* (Darbyshire et al. 2022), *Lumbricus rubellus* (Short et al. 2023), *Lumbricus terrestris* (Blaxter et al. 2023), *Piscicola geometra* (Doe et al. 2023), and *S. limicola* (Darbyshire et al. 2023). Genomes from other sources with accompanying publications are *B. longqiensis* (He et al. 2023), *H. manillensis* (Liu et al. 2023), *M. vulgaris* (Jin et al. 2020), *O. fusiformis* (Martín-Zamora et al. 2023), *P. echinospica* (Sun et al. 2021), *S. benedicti* (Zakas et al. 2022), *S. nudus* (Zheng et al. 2023), and *U. unicinctus* (Cheng et al. 2024).

One species, *O. fusiformis*, had available GenBank gene annotations. Gene prediction for the remaining 23 species was performed using RepeatModeler2 (v2.0.4) (Flynn et al. 2020), RepeatMasker (v4.1.5) (Smit et al. 2015), and the BRAKER3 pipeline (v3.0.3) (Stanke et al. 2006, 2008; Li et al. 2009; Barnett et al. 2011; Lomsadze et al. 2014; Buchfink et al. 2015; Hoff et al. 2016, 2019; Brůna et al. 2021; Gabriel et al. 2024) as reported previously (Lewin et al. 2024b). For species with available RNA-seq data (supplementary table S10, Supplementary Material online), reads were trimmed with fastp (v0.23.4) (Chen et al. 2018) and mapped with STAR (v2.7.10b) (Dobin et al. 2013) before BRAKER3 was run in RNA-seq mode. For species with no RNA-seq data, BRAKER3 was run in protein mode using the supplied Metazoa.fa protein file. Gene prediction quality was assessed using BUSCO (v5.4.7) (Simão et al. 2015). The genome for *Branchellion lobata* was excluded from the main analyses because it has a low genome BUSCO completeness score (72.9% with the metazoan_obd10 database) (Simão et al. 2015) but is included as supplementary fig. S23, Supplementary Material online.

### Phylogenetic Analysis

Single-copy orthologs were identified with OrthoFinder (v2.5.4) (Emms and Kelly 2019). The tree splitting and pruning algorithm of OrthoSNAP (v0.0.1) (Steenwyk et al. 2022) was then used to recover additional single-copy orthologs from gene family trees. Sequences of each ortholog were aligned with MAFFT (v7.520) (Katoh et al. 2002; Katoh and Standley 2013), trimmed with ClipKIT (v1.4.1) (Steenwyk et al. 2020), and concatenated with PhyKIT (v1.11.7) (Steenwyk et al. 2021), before maximum likelihood phylogeny inference with IQ-TREE (v2.2.2.3) (Minh et al. 2020). ModelFinder (Kalyaanamoorthy et al. 2017) was used for automatic substitution model selection, and UFBoot2 (Hoang et al. 2018) was used to perform 1,000 ultrafast bootstrap replicates.

### Macrosynteny Analysis

SyntenyFinder (Lewin et al. 2024a) was used to implement OrthoFinder (Emms and Kelly 2019) and RIdeogram (v0.2.2) (Hao et al. 2020) and produce Oxford dot plots. Bilaterian ALGs were determined by orthology (Simakov et al. 2022). Unless stated otherwise, links between chromosomes with fewer than ten shared orthologs are trimmed from ribbon plots for clarity; all genes are shown in Oxford dot plots.

### WGD Inference

Four complementary methods were used to test for WGDs. First, structural information was inferred from idiogram plots and Oxford dot plots using single-copy orthologs as above. Second, structural information was inferred from Oxford dot plots using multicopy orthologs, permitting up to five paralogs in one species. In both methods 1 and 2, repeated cases of a one:many ratio of genome regions in one species versus another are suggestive of WGD. Third, *K_S_* plots showing distributions of synonymous substitutions per synonymous site were produced for paralogs within annelid genomes using “wgd dmd” and “wgd ksd” in wgd (v2.0.26) (Zwaenepoel and Van de Peer 2019; Chen and Zwaenepoel 2023). *K_S_* measures the divergence of sequences, and, assuming neutral evolution and a constant rate of change, the *K_S_* between two paralogs is an estimate of the age of the duplication. If no WGD is present, the number of genes with a given *K_S_* is expected to decrease exponentially as *K_S_* increases (Lynch and Conery 2003). WGDs generate many duplicates simultaneously, creating duplicates with a similar *K_S_* value and resulting in a peak in the plot. Fourth, blocks of genes with conserved microsynteny were identified within annelid genomes using “wgd syn” (Zwaenepoel and Van de Peer 2019; Chen and Zwaenepoel 2023). The presence of many pairs of gene blocks with conserved microsynteny is suggestive of WGD.

### Rearrangement Index

A “rearrangement index” (*R_i_*) was developed to quantify the extent to which ALG rearrangement has occurred in bilaterian genomes. For each ALG, the rearrangement index is calculated as follows:


(1) $$R_{\mathrm{ALG}}\;=1-(S_{\mathrm{CHR}\;}\times \;C_{\mathrm{CHR}}),$$

where *R*_ALG_ denotes the rearrangement index for a given ALG, *S*_CHR_ (ALG splitting parameter) represents the highest proportion of genes from this ALG on a single chromosome, and *C*_CHR_ (ALG combining parameter) is the proportion of genes on that chromosome that belong to that particular ALG. By incorporating these parameters, the index accounts for both ALG splitting and ALG combining.

Subsequently, the *R_i_* for each genome is given by the equation:


(2) $$R_{i}\;=\;\frac{\Sigma \;(R_{\mathrm{ALG}})}{N},$$

where *R_i_* denotes the rearrangement index for the genome, *R*_ALG_ the is rearrangement index for each ALG, and *N* is the total number of ALGs. The higher the index, the higher the level of interchromosomal rearrangements. It is important to note that the index serves as a general indicator of the level of rearrangement in a given genome. Therefore, minor differences between species should be interpreted with caution.

For Fig. 5b, we plot the ALG splitting index (*S_i_*) where *S*_ALG_ = 1 − *S*_CHR_ and *S_i_* = Σ(*S*_ALG_)/*N*; and the ALG combining index (*C_i_*) where *C*_ALG_ = 1 − *C*_CHR_ and *C_i_* = Σ(*C*_ALG_)/*N*.

### RNA-seq Data Analysis

Raw RNA-sequencing data (supplementary table S11, Supplementary Material online) from *M. vulgaris* tissues were downloaded from NCBI SRA using SRA toolkit (Leinonen et al. 2011) and GNU parallel (v20230322) (Tange 2023). Gene expression was quantified with the pseudo-aligner salmon (v1.10.2) (Patro et al. 2017).

### Statistical Analysis

Statistical analysis was performed using R (v4.3.0) (R Core Team 2023). Spearman's rank correlation was used to test whether the number of fusions of each ALG correlates with the average length of chromosomes on which the ALG is hosted, as described previously (Wright et al. 2024). Chromosome length was measured as a proportion of the total genome length. A *χ*^2^ test was used to test for differences in the number of fusions per ALG. Linear models were used to examine the relationship between rearrangement index and chromosome number. *P* < 0.05 was considered to be statistically significant.

## Supplementary Material

msae172_Supplementary_Data

## Data Availability

Gene models, including coding sequences (*.fasta), protein sequences (*.faa), and gene annotations in gene transfer format (GTF) (*.gtf) for 23 annelid species annotated in this study, have been deposited in Dryad (https://doi.org/10.5061/dryad.brv15dvhv). Custom Python script used for calculating genome rearrangement index is available in our GitHub repository (https://github.com/symgenoevolab/RearrangementIndexer).
